# Mitochondrial Transplantation Restores Immune Cell Metabolism in Sepsis: A Metabolomics Study

**DOI:** 10.3390/ijms27010332

**Published:** 2025-12-28

**Authors:** Tae Nyoung Chung, Se Rin Choi, Su-Hyun Kim, Choong Hwan Lee, Kyuseok Kim

**Affiliations:** 1 Department of Emergency Medicine, CHA University School of Medicine, 59 Yatap-Ro, Bundang-gu, Seongnam-si 13496, Republic of Korea; hendrix74@cha.ac.kr; 2 Department of Bioscience and Biotechnology, Konkuk University, Seoul 05029, Republic of Korea; csr0701@gmail.com (S.R.C.); kimsuhyun2019@naver.com (S.-H.K.)

**Keywords:** sepsis, mitochondrial transplantation, immune cells, peripheral blood mononuclear cells, splenocytes, metabolomics, gas chromatography–mass spectrometry, energy metabolism, oxidative phosphorylation, mitochondrial dysfunction

## Abstract

Sepsis induces severe immune and metabolic dysfunction driven by mitochondrial failure. Mitochondrial transplantation (MT) has emerged as a promising strategy to restore mitochondrial bioenergetics, but its metabolic impact on immune cells remains unclear. Here, we used gas chromatography–time-of-flight mass spectrometry (GC-TOF-MS)-based metabolomics to evaluate metabolic alterations in peripheral blood mononuclear cells (PBMCs) and splenocytes from a rat polymicrobial sepsis model treated with MT. Principal component and partial least-squares discriminant analyses revealed distinct clustering between sham, sepsis, and MT groups. Sepsis markedly suppressed metabolites related to amino acid, carbohydrate, and lipid metabolism, including aspartic acid, glutamic acid, AMP, and myo-inositol, reflecting mitochondrial metabolic paralysis. MT partially restored these metabolites toward sham levels, reactivating tricarboxylic acid (TCA) cycle, nucleotide, and lipid pathways. Pathway analysis confirmed that exogenous mitochondria reversed sepsis-induced metabolic suppression and promoted bioenergetic recovery in immune cells. These findings provide direct metabolomic evidence that MT reprograms immune metabolism and restores oxidative and biosynthetic function during sepsis, supporting its potential as a mitochondrial-based metabolic therapy.

## 1. Introduction

Sepsis is a life-threatening condition characterized by a dysregulated host response to infection that frequently leads to metabolic collapse and organ failure [1,2]. The global burden of sepsis has been increased, and mortality rate is very high [3,4].

Beyond its systemic inflammatory features, sepsis is increasingly recognized as a disease of profound immunometabolic dysregulation [1]. Effective immune responses require coordinated metabolic remodeling to meet the energetic and biosynthetic demands of activation. In sepsis, however, immune cells fail to maintain this metabolic flexibility, resulting in impaired tricarboxylic acid (TCA) cycle activity, amino acid depletion, and disrupted nucleotide and lipid metabolism [5]. Peripheral blood mononuclear cells (PBMCs) and splenocytes represent metabolically active immune compartments that are particularly susceptible to such metabolic stress [6,7,8]. This emerging immunometabolic perspective provides a conceptual framework for understanding how mitochondrial dysfunction contributes to immune paralysis in sepsis.

At the cellular level, this immunometabolic failure is largely driven by mitochondrial dysfunction, which impairs energy metabolism and exacerbates oxidative stress [9]. Previous studies have shown that mitochondrial impairment in immune cells is associated with immune paralysis and poor outcomes in sepsis patients [10,11].

Mitochondrial transplantation (MT) has emerged as a promising therapeutic approach to restore cellular bioenergetics and metabolism in injured or dysfunctional tissues [12,13]. Recent preclinical studies have explored MT as a therapeutic strategy to restore mitochondrial function and cellular metabolism in sepsis [14,15]. MT involves the delivery of viable mitochondria into dysfunctional cells to re-establish mitochondrial respiration and adenosine triphosphate (ATP) production [16]. Although encouraging results have been reported in various models including cardiac ischemia and liver injury, the metabolic outcomes of MT in sepsis remain to be elucidated.

Metabolomics offers a powerful approach to capturing the systemic metabolic changes in disease states [17]. In this study, we employed gas chromatography-time-of-flight-mass spectrometry (GC-TOF-MS)-based metabolomics to assess the metabolic perturbations induced by sepsis in murine PBMCs and splenocytes, and to evaluate the therapeutic impact of MT. Our findings provide insights into the metabolic reprogramming associated with sepsis and suggest the potential of MT to restore immune cell metabolism.

## 2. Results

### 2.1. Quality Control of Isolated Mitochondria

To verify the quality of mitochondria isolated from L6 cells, we evaluated their yield, purity, and functional integrity. The isolation procedure yielded approximately 1063 μg of mitochondrial protein from 6 × 10^7^ cells (Figure S1A). Flow cytometric analysis using dual staining with MitoTracker Green-FITC and the outer mitochondrial membrane marker Tomm20-APC demonstrated a high purity of 98.77% (Figure S1B), indicating successful enrichment of the mitochondrial fraction with minimal contamination by cellular debris. Furthermore, functional integrity of the isolated mitochondria was confirmed by a preserved membrane potential, with 98.04% of mitochondria exhibiting TMRE positivity (Figure S1C). Consistent with these findings, the isolated mitochondria showed substantial bioenergetic activity, maintaining an ATP synthesis level of 769 nM (Figure S1D). Collectively, these results demonstrate that the isolated mitochondria were highly pure and functionally competent, supporting their suitability for subsequent downstream analyze.

### 2.2. Global Metabolic Profile Distinction

**Figure 1:**
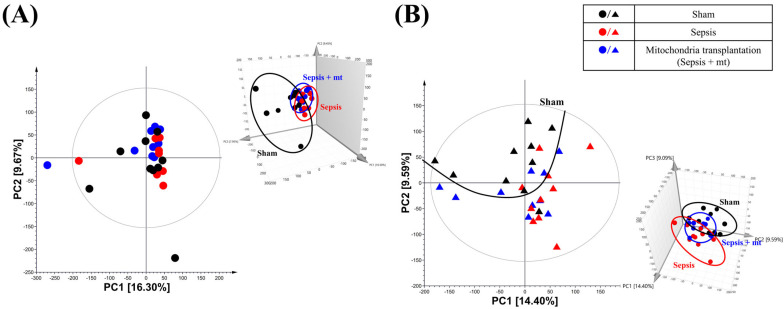
Principal component analysis (PCA) score plots of metabolites in peripheral blood mononuclear cells (PBMCs) and splenocytes. PCA score plots derived from gas chromatography–time-of-flight mass spectrometry metabolomic data showing the global metabolic distribution among the sham, sepsis, and mitochondrial transplantation (MT) groups. (**A**) Peripheral blood mononuclear cells (PBMCs) and (**B**) splenocytes display distinct clustering patterns, indicating clear metabolic separation between the experimental groups.

**Figure 2:**
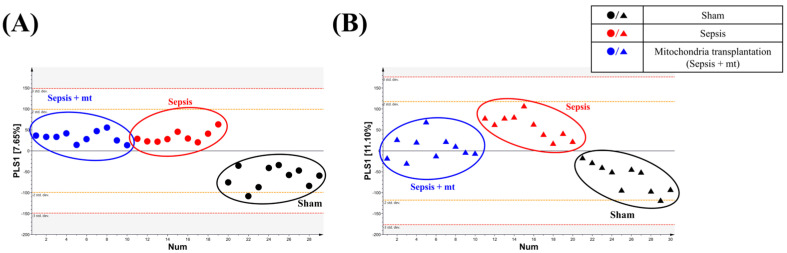
Partial least-squares discriminant analysis (PLS–DA) score plots of peripheral blood mononuclear cells (PBMCs) and splenocytes. PLS–DA models illustrating the metabolic discrimination between sham, sepsis, and MT groups. (**A**) PBMCs and (**B**) splenocytes show distinct separation along the first principal component (PLS1), confirming significant metabolic shifts induced by sepsis and partial recovery following mitochondrial transplantation.

Samples analyzed using GC-TOF-MS were used for multivariate analysis of each feature. After that, by comparing the integrated metabolites, the relative abundance of metabolites for each cell group was quantified. We performed principal component analysis (PCA) and partial least-squares discriminant analysis (PLS-DA) for evaluating differences and consistencies in the metabolite profiles of sham, sepsis and mitochondrial transplantation groups in PBMCs and splenocytes (Figure 1 and Figure 2).

In the two-dimensional (2D) PCA plot, a clear separation among the three groups (sham, sepsis, and sepsis with MT) was not observed; however, the three-dimensional (3D) plot demonstrated a tendency toward clustering (Figure 1). In the PLS-DA analysis, PBMC samples showed a trend in which the sham group was separated from the sepsis and sepsis with MT groups along the PLS1 axis, although this difference was not statistically significant (*p* = 0.16, Figure 2A). In contrast, splenocytes displayed a statistically significant separation among the groups (*p* = 0.04, Figure 2B). The results indicate that sepsis caused significant differences in metabolite levels.

### 2.3. Heatmap Analysis Associated with the Altered Metabolites

**Figure 3:**
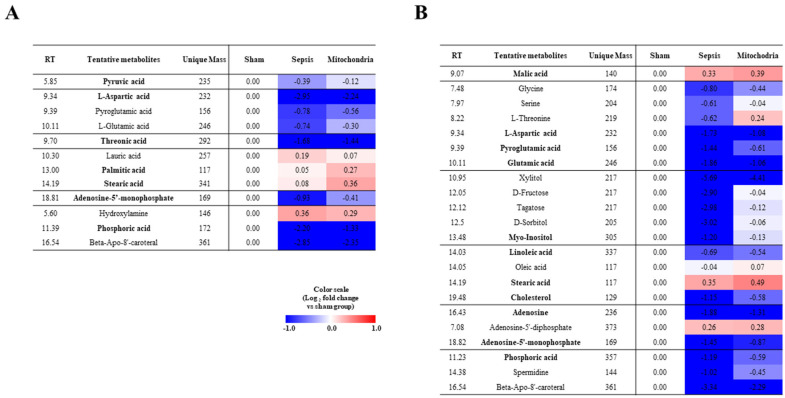
Heatmap visualization of altered metabolites in peripheral blood mononuclear cells (PBMCs) and splenocytes. Heatmaps of altered metabolites identified by gas chromatography–time-of-flight mass spectrometry in (**A**) PBMCs and (**B**) splenocytes. Color intensity represents the fold change of each metabolite relative to the sham group. Metabolites showing significant differences among groups were indicated in bold (one-way ANOVA, *p* < 0.05). Most amino acids, sugar derivatives, and fatty acids were downregulated in sepsis compared to the sham group and were partially restored after mitochondrial transplantation.

The PBMC samples exhibited differences in metabolites for each group. Furthermore, most metabolites exhibited a higher relative abundance under the sham group (Figure 3A). Compared to sepsis and mitochondrial transplantation groups, the levels of amino acids, sugar derivatives, and fatty acids were higher in the mitochondrial transplantation group than sepsis group. Especially, the relative content of aspartic acid decreased rapidly in the sepsis group compared to the sham group. It tends to recover to some extent after mitochondrial transplantation. The splenocytes samples exhibited similarity in metabolite contents with PBMC samples (Figure 3B).

### 2.4. Effect of Sepsis and Mitochondrial Transplantation on the Metabolic Pathways

**Figure 4:**
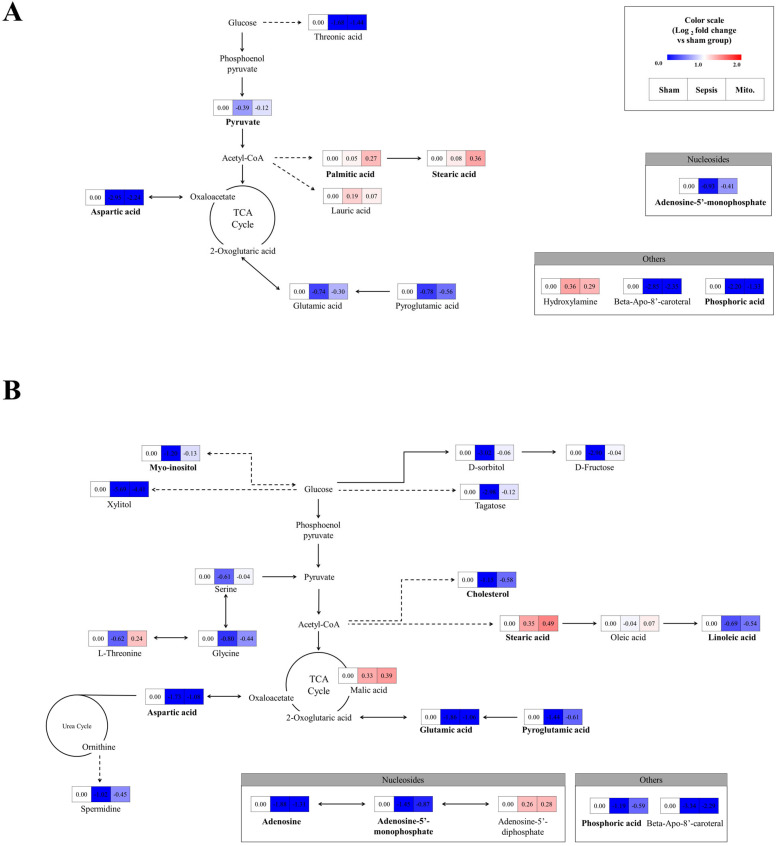
Metabolic pathway alterations associated with mitochondrial transplantation in sepsis. Pathway mapping of discriminant metabolites showing the impact of sepsis and the restorative effects of mitochondrial transplantation (MT) in (**A**) peripheral blood mononuclear cells and (**B**) splenocytes. Metabolites downregulated in sepsis and recovered after MT are highlighted, including intermediates of amino acid metabolism, the tricarboxylic acid (TCA) cycle, and carbohydrate metabolism. These results indicate that exogenous mitochondria promote the reactivation of oxidative and biosynthetic metabolic pathways in immune cells.

To visualize the effect of mitochondrial transplantation in sepsis on metabolites more clearly, we performed pathway analysis with altered metabolites of PBMCs and splenocytes. The identified pathways are shown in Figure 4. In both PBMCs and splenocytes, pathway analysis revealed that sepsis predominantly suppressed central carbon metabolism, including amino acid metabolism, carbohydrate metabolism, and the TCA cycle. Notably, pathways related to alanine, aspartate, and glutamate metabolism, as well as glycolysis and pyruvate metabolism, showed marked downregulation in the sepsis group. Following mitochondrial transplantation, these pathways exhibited partial but consistent recovery toward sham levels, indicating reactivation of mitochondrial-dependent metabolic fluxes. The coordinated restoration of multiple interconnected pathways suggests that mitochondrial transplantation does not affect isolated metabolites but rather promotes a global reorganization of intracellular metabolic networks in immune cells.

## 3. Discussion

This study provides a metabolomics-based overview of the metabolic alterations caused by sepsis and the restorative effects of MT on immune cells, particularly PBMCs and splenocytes. Using GC-TOF-MS, we identified that sepsis induced broad suppression of metabolites involved in amino acid, carbohydrate, and lipid metabolism, while MT partially restored these metabolic signatures toward those of the sham group. These findings highlight the potential of MT as a metabolic therapy for restoring immune homeostasis in sepsis.

Sepsis is characterized by mitochondrial dysfunction that leads to impaired oxidative phosphorylation (OXPHOS), ATP depletion, and increased oxidative stress [18,19]. Consistent with these mechanisms, our metabolomic analysis revealed significant reductions in key metabolites such as aspartic acid, glutamic acid, AMP, myo-inositol, and cholesterol in septic PBMCs and splenocytes. Aspartic and glutamic acids are integral intermediates in the TCA cycle and amino acid biosynthesis, and their depletion suggests inhibition of mitochondrial respiration and anaplerotic flux. Likewise, the reduction of pyruvic acid implies a limitation of glycolytic input into the TCA cycle, likely caused by pyruvate dehydrogenase inactivation, which is well-documented in sepsis-associated metabolic paralysis [5,20].

In our results, most of these metabolites were partially restored to levels comparable to those of the sham group following MT. The recovery of aspartic acid, glutamic acid, and adenosine monophosphate (AMP) indicates reactivation of mitochondrial oxidative metabolism and improved nucleotide biosynthesis [21,22]. Furthermore, normalization of myo-inositol and stearic acid implies restoration of membrane lipid turnover and redox balance [23,24,25]. In splenocytes, additional recovery of carbohydrate-related metabolites such as fructose, glucitol, and tagatose reflects renewed glycolytic and pentose phosphate pathway activity [26,27,28]. Collectively, these observations suggest that exogenous mitochondria restore metabolic flexibility in immune cells, a key determinant for reversing immune exhaustion in sepsis.

The beneficial effects of MT in sepsis have been reported in previous studies. Hwang et al. [14] showed that MT attenuated apoptosis and restored splenic immune function in polymicrobial sepsis. Similarly, Kim et al. [29] demonstrated that the efficacy of MT depends on the cellular source of donor mitochondria, improving ATP production and survival across multiple cell types in septic models. Our findings align with these studies, supporting the concept that MT exerts immunometabolic benefits by replenishing mitochondrial capacity. However, while those studies mainly assessed survival, inflammation, and respiratory parameters, our current work provides direct metabolic evidence at the level of amino acid, carbohydrate, and lipid pathways.

Previous metabolomics studies have also revealed that sepsis reduces circulating amino acids (e.g., tryptophan, phenylalanine, glutamate), purine intermediates, and sugar alcohols [30,31,32]. Most of these studies analyzed plasma or urine, which cannot delineate cell-type–specific metabolic vulnerabilities. By separately profiling PBMCs and splenocytes, our study reveals that MT affects distinct metabolic pathways in different immune compartments—predominantly amino acid and nucleotide metabolism in PBMCs and carbohydrate metabolism in splenocytes—suggesting that metabolic recovery patterns are cell-type dependent. This finding complements Kim et al. [29], who emphasized donor cell-type variability, by demonstrating that host immune cell identity also dictates the metabolic response to MT.

In the present study, imipenem was used as an antibiotic during the establishment of the sepsis model. Several in vitro studies have reported that high doses of imipenem can impair mitochondrial function in certain cell types [33,34], suggesting a potential influence on our study, which involved mitochondrial administration. However, these effects were observed only at high concentrations and were limited to specific cell types; thus, the likelihood of a significant impact in our experimental setting is considered low. Nevertheless, future investigations should evaluate whether the use of such antibiotics may affect the function of externally administered mitochondria.

Our results also converge with the emerging paradigm of immunometabolic reprogramming in sepsis. Previous reports have shown that immune cells in sepsis shift toward glycolysis at the expense of oxidative phosphorylation, a “Warburg-like” metabolic transition that leads to immune dysfunction [18,19]. The partial restoration of TCA and amino acid metabolism after MT suggests that exogenous mitochondria can reverse this metabolic lock, reinstating oxidative metabolism and biosynthetic capacity. Recent review has proposed that mitochondrial transfer between cells can modulate immune responses and reduce exhaustion [35]. Our in vivo data provide direct biochemical support for these mechanistic hypotheses. Among the potential mechanisms of mitochondrial transplantation identified in recent studies, one proposed mechanism is that transplanted mitochondria function as scavengers of excessive calcium ions [36,37]. Given the well-documented association between excessive calcium ions and increased mortality or organ dysfunction in sepsis [38,39], the therapeutic effects of MT on sepsis observed in the present study are likely mediated, at least in part, by its capacity to buffer and scavenge excessive calcium ions. However, intracellular calcium levels were not directly measured in the present study, and no previous studies have directly demonstrated calcium scavenging by transplanted mitochondria in sepsis. Therefore, future studies incorporating direct calcium measurements will be necessary to validate this proposed mechanism.

The present study adds several new insights to the growing body of MT research.

First, it extends previous functional observations by providing metabolite-level evidence of MT-mediated recovery in sepsis. Earlier studies primarily quantified mitochondrial respiration, reactive oxygen species (ROS) levels, or apoptosis [14,29]; here, we identify specific metabolites (e.g., aspartate, AMP, myo-inositol) reflecting reactivation of TCA, nucleotide, and lipid metabolism after MT.

Second, the consistent metabolic trends observed in both PBMCs and splenocytes demonstrate that MT exerts a systemic metabolic reprogramming effect rather than a localized correction, supporting the concept that mitochondrial dysfunction is a global driver of immune dysregulation [19].

Third, compared with pharmacological approaches targeting mitochondrial biogenesis or antioxidant capacity, MT directly replenishes functional organelles, promoting a more comprehensive normalization of metabolic pathways.

Our findings also support recent recommendations that metabolic profiling should accompany mitochondrial-based therapies to verify their systemic and bioenergetic effects [40]. Therefore, this study serves as a preclinical framework for integrating metabolomics into the evaluation of MT in sepsis and related diseases.

Despite the clear metabolic distinctions revealed, several limitations warrant discussion. The present study focused on metabolomic profiling without direct measurements of mitochondrial respiration, ROS production, or immune activation. In addition, due to technical constraints, the extent of mitochondrial transplantation could not be quantitatively assessed in individual animals, precluding correlation analyses between mitochondrial uptake efficiency and metabolomic profiling data. Integrating metabolomics with transcriptomic and proteomic analyses would provide a more complete understanding of the regulatory networks linking mitochondrial uptake to immune recovery. Furthermore, longitudinal analyses are needed to determine whether metabolic restoration correlates with survival or organ protection. Future work should also investigate the mechanisms of mitochondrial internalization, persistence, and bioenergetic integration in immune cells.

## 4. Materials and Methods

### 4.1. In Vivo Sepsis Model

All animal procedures were conducted following approval from the Institutional Animal Care and Use Committee (IACUC) of CHA University (IACUC No. 230143) and in accordance with the National Institutes of Health guidelines for the care and use of laboratory animals. The study was also performed in compliance with the Animal Research Reporting of In Vivo Experiments (ARRIVE) guidelines. Male Sprague–Dawley rats weighing 270–330 g were housed under controlled environmental conditions (temperature 20–24 °C) with free access to standard chow and water for at least seven days prior to experimentation.

A body weight-adjusted polymicrobial sepsis model was established based on previously described methods, using the cecal slurry model [41]. Briefly, donor rats were anesthetized with intramuscular injections of Zoletil (50 mg/kg) and xylazine (10 mg/kg). After a mid-line laparotomy, the cecum was exteriorized, and a 0.5 cm incision was made along the antimesenteric border to expel fecal material. Donor animals were then euthanized, and the collected feces were weighed and diluted with 5% dextrose saline at a 1:3 ratio. Prior to sepsis induction, recipient rats were anesthetized as described above. Following a small midline laparotomy, the homogenized fecal slurry was administered into the peritoneal cavity. The total injected volume was adjusted according to each animal’s body weight (5.5 mL/kg). Afterward, subcutaneous fluid resuscitation was provided using 5% dextrose saline (30 mL/kg), and imipenem (25 mg/kg) was administered subcutaneously twice daily for two consecutive days.

Animals were randomly allocated into three groups (n = 10 per group): a sham group, a sepsis-induced group, and a sepsis induction with MT group. Randomization was performed using the random number generation function in Excel (Microsoft Corporation, Redmond, WA, USA). Metabolomic analysis was conducted in a laboratory separate from the one where the animal procedures, including sepsis induction and MT administration, were performed. Each sample was assigned an independent identification code to ensure that the analyst was blinded to the experimental group information. In the sham group, the animals underwent laparotomy in the same manner as the sepsis-induced animals, but the abdomen was closed without the administration of fecal slurry. One hour after the procedure, MT or DBPS (200 µg) was administered via the tail vein, following previously described methods [14]. After 24 h, PBMCs/splenocytes were isolated for metabolomic analysis. Animals that did not survive until the scheduled endpoint were excluded from analysis. During the observation period, animal facility staff monitored the rats twice daily. Moribund animals were humanely euthanized according to veterinary assessment.

### 4.2. Mitochondria Isolation and Quality Control

We isolated mitochondria as previously described [14]. Briefly, L6 cells (ATCC; CRL-1458, Manassas, VA, USA) were homogenized using a 26G syringe in SHE buffer (0.25 M sucrose, 20 mM HEPES [pH 7.4], 2 mM EGTA, 10 mM KCl, 1.5 mM MgCl_2_, and 0.1% defatted BSA with protease inhibitors), followed by centrifugation at 1500× *g* for 5 min at 4 °C. The supernatant was then centrifuged at 20,000× *g* for 10 min to obtain mitochondria.

The quality of the isolated mitochondria was evaluated using protein quantification, flow cytometry, and functional assays. The total protein yield of the isolated mitochondrial fraction was quantified using the Pierce bicinchoninic acid protein assay kit (Thermo Scientific, Waltham, MA, USA). Mitochondrial purity and structural integrity were assessed by CytoFLEX flow cytometry (Beckman Coulter, CA, USA) following dual staining with MitoTracker Green (Invitrogen, Waltham, MA, USA) and an APC conjugated-Tomm20 (Abcam, Cambridge, UK). Functional viability was determined by measuring mitochondrial membrane potential (MMP) using tetramethylrhodamine ethyl ester (TMRE; Invitrogen, Waltham, MA, USA) staining. Finally, mitochondrial bioenergetic capacity was validated by quantifying ATP synthesis levels using the CellTiter-Glo 2.0 (Promega, Madison, WI, USA) reagent, following the manufacturer’s protocol.

### 4.3. Metabolomics Study

#### 4.3.1. Extraction of Animal Samples for Metabolomics

For the isolation of PBMCs, 9–10 mL of whole blood was collected from the abdominal aorta of each animal and subjected to density gradient centrifugation over Ficoll-Paque Plus (GE Healthcare, Marlborough, MA, USA), as previously described [42]. Frozen spleen tissues (100 mg each) were homogenized in 1 mL of 100% methanol containing the same internal standard, as described elsewhere [43]. The mixtures were processed using a Retsch MM400 mixer mill (Retsch GmbH & Co., Haan, Germany) at 30 Hz for 10 min. The homogenates were centrifuged at 12,000 rpm for 10 min at 4 °C, and the resulting supernatants were filtered through 0.2 μm PTFE syringe filters. The filtrates were transferred to Eppendorf tubes, completely dried using a speed vacuum concentrator, and stored at −80 °C. Before instrumental analysis, dried extracts were reconstituted in 100% methanol to a final concentration of 10 mg/mL, re-dried under vacuum, and subjected to a two-step derivatization process.

#### 4.3.2. Gas Chromatography-Time-of-Flight-Mass Spectrometry Analysis

GC–TOF–MS analysis was carried out using an Agilent 7890A gas chromatograph coupled with an Agilent 7693 autosampler (Agilent Technologies, Atlanta, GA, USA), as previously described [44]. The dried extracts were first oximated with 50 μL of methoxyamine hydrochloride (20 mg/mL in pyridine) at 30 °C for 90 min in a thermomixer (Eppendorf, Hamburg, Germany). Subsequently, the samples were silylated with 50 μL of N-methyl-N-(trimethylsilyl) trifluoroacetamide (MSTFA) at 37 °C for 30 min. Pooled quality control (QC) samples were prepared by combining 10 μL aliquots from each sample.

A 1 μL aliquot of each derivatized sample was injected in splitless mode. Chromatographic separation was performed on an Rtx-5MS column (i.d., 30 m × 0.25 mm, 0.25 µm particle size; Restek Corp., Bellefonte, PA, USA) using helium gas as a carrier gas at a constant flow rate of 1.5 mL/min. Injector and ion source temperatures were maintained at 250 °C and 230 °C, respectively. The oven temperature was initially held at 75 °C for 2 min, increased to 300 °C at 15 °C/min, and maintained for an additional 3 min. Spectra were recorded at 10 scans per second over a mass range of 50–1000 *m*/*z*, using a detector voltage of 1640 V. Analytical runs were performed in randomized batches of eight samples, interspersed with QC samples to ensure analytical reproducibility and robustness.

### 4.4. Data and Statistical Analysis

Mass Spectrometry (MS) data were processed and analyzed following previously published procedures [44]. Raw spectra were converted into NetCDF (*.cdf) format using ChromaTOF software (v4.44, LECO). The resulting files were processed in MetAlign (https://github.com/nlapier2/Metalign, accessed on 11 October 2025) to extract retention times, accurate masses, and normalized peak intensities, generating a data matrix exported to Microsoft Excel for further analysis.

Multivariate statistical analyses, including PCA and PLS–DA, were conducted using SIMCA-P+ software (v12.0, Umetrics, Umea, Sweden). Discriminant metabolites were identified based on a variable importance in projection (VIP) score > 0.7, and their statistical significance among groups were evaluated using one-way ANOVA (*p* < 0.05).

Metabolites identified through GC–TOF–MS were annotated by comparison with authentic standards, retention times, and mass spectral fragments. Additional confirmation was obtained using in-house spectral libraries and public databases, including the National Institute of Standards and Technology (NIST) Mass Spectral Database (v2.0, 2011, FairCom, Gaithersburg, MD, USA) and the Human Metabolome Database (HMDB; http://www.hmdb.ca/).

## Data Availability

The datasets generated and analyzed during the current study are available from the corresponding author on reasonable request.

## Supplementary Materials

The following supporting information can be downloaded at: https://www.mdpi.com/article/10.3390/ijms27010332/s1.
