# Supplementary material for: Mitochondrial Transplantation Restores Immune Cell Metabolism in Sepsis: A Metabolomics Study
Source: Int J Mol Sci. 2025 Dec 28;27(1):332. doi: 10.3390/ijms27010332 (PMC12785550; doi:10.3390/ijms27010332)
Supplement: Supplementary file 1 [file ijms-27-00332-s001.zip › ijms-4031554-supplementary.pdf]

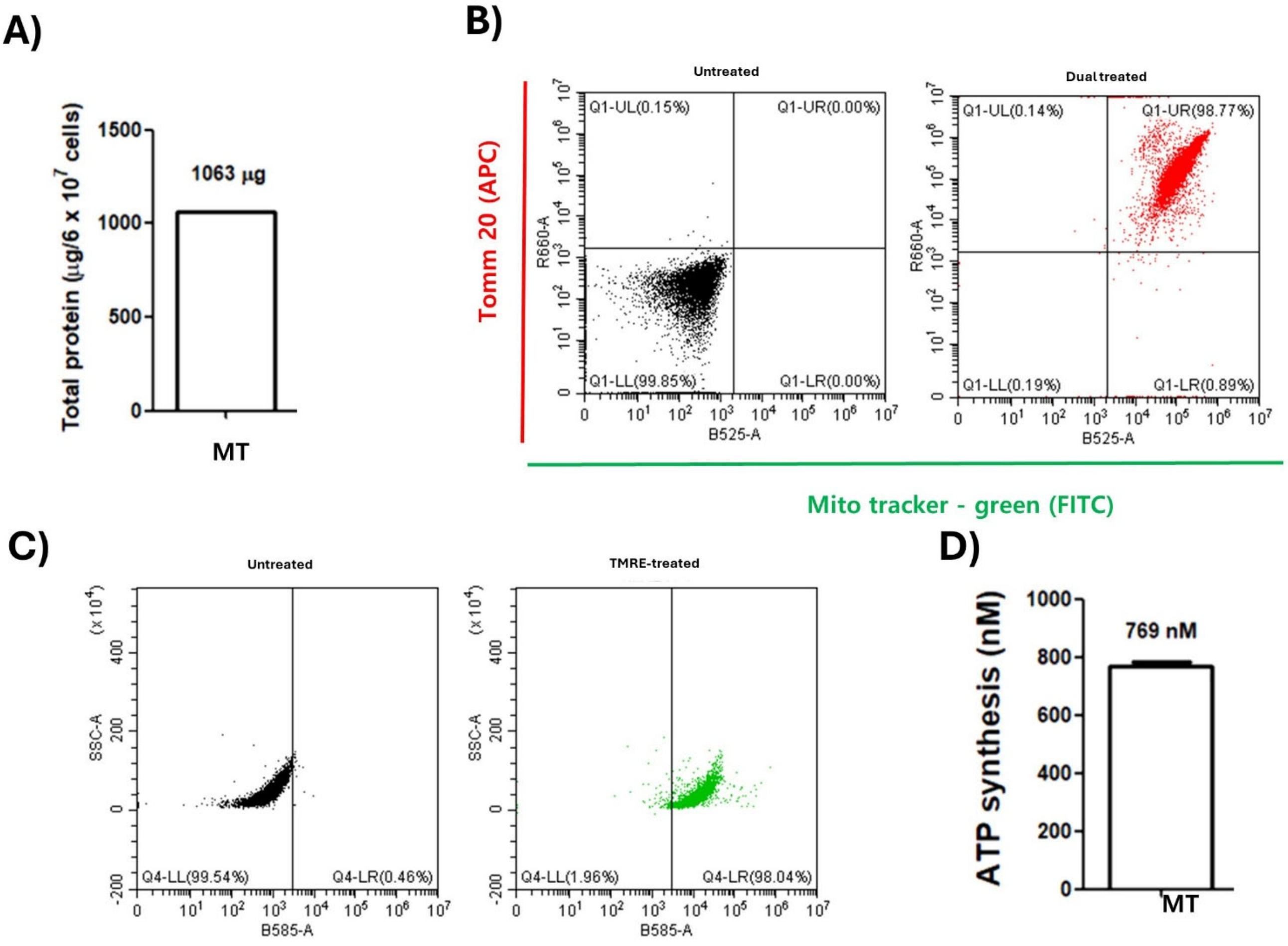

Figure S1. Quality Control and Functional Validation of Mitochondria Isolated from L6 Cells. A) Total mitochondrial protein yield quantified by BCA assay, showing a recovery of 1,063 μg from 6 X 10^7 cells. B) Flow cytometric assessment of mitochondrial purity. Dual staining with MitoTracker Green (FITC) and Tomm20 (APC) revealed a high-purity population of 98.77% (Q1-UR). C) Evaluation of mitochondrial membrane potential (MMP) via TMRE staining. Approximately 98.04% of the isolated mitochondria maintained active MMP. D) Bioenergetic function as measured by ATP synthesis capacity, showing a production level of 769 nM.
